# Linking appraisal to behavioral flexibility in animals: implications for stress research

**DOI:** 10.3389/fnbeh.2015.00104

**Published:** 2015-04-27

**Authors:** Ana I. Faustino, Gonçalo A. Oliveira, Rui F. Oliveira

**Affiliations:** ^1^Integrative Behavioral Biology Laboratory, Instituto Gulbenkian de CiênciaOeiras, Portugal; ^2^ISPA Instituto UniversitárioLisboa, Portugal; ^3^Champalimaud Neuroscience Programme, Champalimaud Centre for the UnknownLisbon, Portugal

**Keywords:** behavioral flexibility, appraisal, animal behavior, cognitive bias, stress

## Abstract

In fluctuating environments, organisms require mechanisms enabling the rapid expression of context-dependent behaviors. Here, we approach behavioral flexibility from a perspective rooted in appraisal theory, aiming to provide a better understanding on how animals adjust their internal state to environmental context. Appraisal has been defined as a multi-component and interactive process between the individual and the environment, in which the individual must evaluate the significance of a stimulus to generate an adaptive response. Within this framework, we review and reframe the existing evidence for the appraisal components in animal literature, in an attempt to reveal the common ground of appraisal mechanisms between species. Furthermore, cognitive biases may occur in the appraisal of ambiguous stimuli. These biases may be interpreted either as states open to environmental modulation or as long-lasting phenotypic traits. Finally, we discuss the implications of cognitive bias for stress research.

## Introduction

According to classic evolutionary theory, the modification of behavior by means of natural selection is possible through its action on the genetic components of behavioral variation. Using Mayr (1974) terminology, these genetic programs can be classified as closed or open, depending on their permissiveness to modifications as they are translated into a behavioral program (phenotype). The closed genetic programs represent genetically controlled fixed responses to environmental cues (e.g., fixed action patterns), that have been selected due to the adaptive reliability of a specific response to a stimulus. On the other hand, open programs, by allowing the integration of additional inputs, are therefore subject to environmental modulation during the lifetime of the organism (Mayr, 1974). Thus, behavioral flexibility, which can be seen as a particular case of phenotypic plasticity, permits a rapid pathway for the adjustment to environmental changes that exceeds the rate of evolutionary genetic change (Frank, 2011). Behavioral flexibility is characterized by variable (within and between individuals), reversible, non-cyclic transformations in the individual’s behavior, leaving open the possibility of further adjustments in the organism physiology and morphology (Piersma and Drent, 2003; Taborsky and Oliveira, 2012).

Following previous work (Shettleworth, 2001; Oliveira, 2013), it is known that the range of behavioral flexibility allowed by genetic programs is insufficient for a context-dependent adjustment of behavior to highly variable and complex environments (e.g., social contexts), therefore a greater degree of behavioral flexibility may be understood by accounting for the cognitive abilities of the organism. Using appraisal theory (e.g., Moors et al., 2013), we hypothesize that the capacity of an organism to regulate its internal state according to the evaluation of the conditions of the outside world, rather than using simple stimulus-response processes, is essential to the understanding of behavioral flexibility and the adjustment of organisms to changing environments. In this regard, we explore the study of cognitive biases as resulting from the interference of affective states (Mendl et al., 2009), genetic and environmental factors (Enkel et al., 2010) on the evaluation of ambiguous stimuli, and how they generate different behavioral phenotypes that diverge in their resilience to stressful events.

## The Concept of Appraisal

Appraisal can be defined as an inherently transactional process between the individual and the environment, in which the significance of the event must be detected and assessed by the appraiser (Smith and Kirby, 2009; Moors et al., 2013). Moreover, appraisal is not simply determined by the objective characteristics of the stimuli or the individual’s dispositional characteristics (Scherer, 2001). Instead it results from an interaction, open to the individual’s motivational and physiological state, in which the implications of the stimulus circumstances as they relate to the individual’s needs, resources and abilities are evaluated (Roseman and Smith, 2001; Scherer, 2009; Smith and Kirby, 2009). A key point in appraisal theory is to conceive appraisal not as a single event triggered by environmental or internal changes, but as a dynamic recursive process of appraisal followed by reappraisal, reflecting the constant inflow of information characteristic of changing environments, that must be monitored to correct previous evaluations and generate flexible responses (Scherer, 2001, 2009). Starting from Arnold’s theory (Arnold, 1960) up to the current models (reviewed in Moors et al., 2013), appraisal has been encompassing both automatic and deliberate processes. Therefore, although Lazarus’s work (Lazarus, 1991) refers to appraisal as “cognitive appraisal” given the focus on more controlled processes—this nomenclature should not be interpreted as a denial of the involvement of automatic processes in appraisal (Lazarus, 2001). Accordingly, based on an innovative proposal by Leventhal and Scherer (1987) for appraisal processing in humans, we review its existence in non-human animals through the description and operationalization of the relevant appraisal components performed at different levels of processing (an updated model can be found in Scherer, 2009).

## Appraisal in Animals

Appraisal components (see Table 1, for examples in animal studies) allow the evaluation of an event, by combining both the individual’s affective state and the momentary environmental conditions as contributing factors to the appraisal process (Scherer, 2001; Ellsworth and Scherer, 2003). In this mini-review, we discuss evidence from animal research for specific appraisal components, namely novelty, predictability, pleasantness and coping. Other appraisal components described in humans, like internal (Schomerus et al., 2012) and external standards (Moscovitch and Hofmann, 2007) will not be considered, since these components refer to the influences of self and social-norms in the appraisal process and thus may not be present in non-human animals.

**Table 1:** **Relevant examples of appraisal components in animals**.

Appraisal goal	Appraisal component	Function	Animal	Reference
Relevance	Novelty	Orienting,	Fowl	Dawkins (2002)
		Focusing,	Lamb	Désiré et al. (2002)
		Alerting	Zebrafish	Wong et al. (2010)
	Predictability	Alerting,	Rat	van den Bos et al. (2003)
		Readiness,	Rat	Weiss (1970)
		Anticipation	Cichlid fish	Galhardo et al. (2011)
			Zebrafish	Piato et al. (2011)
			Lamb	Greiveldinger et al. (2007)
			Fowl	Zimmerman et al. (2011)
	Pleasantness	Approach,	Zebrafish	Xu et al. (2007)
		Withdrawal	Zebrafish	Al-Imari and Gerlai (2008)
			Sea bream	Millot et al. (2014)
Implication and Coping	Coping	Control,	Rat	Weiss (1968)
		Adjustment	Rat	Overmier et al. (1980)
			Lamb	Greiveldinger et al. (2009)

A set of appraisal components (novelty, predictability and pleasantness) rely on the organism’s ability to determine if the stimulus is significant enough to elicit further processing (i.e., stimulus relevance, Ellsworth and Scherer, 2003). When an individual is confronted with a novel event (novelty component), its response will be determined by stimulus suddenness and salience (Ellsworth and Scherer, 2003). In zebrafish, for example, changes in exploratory behavior in response to a novel tank have been described, such that fish responded with an increase in exploratory swimming and a decrease in freezing within the first minutes of the test (Wong et al., 2010). Moreover, over the course of 7 days of daily exposures to the same tank, fish habituated to the tank, that is they spent more time in exploration and less time in freezing, a reflection of acquired familiarity with the stimulus. Besides familiarity, an animal is also able to detect regularities in the environment (e.g., association between stimuli), in order to estimate the probability of a certain event occurring—predictability component (Ellsworth and Scherer, 2003). The anticipation of rewarding and aversive stimuli allows animals the ability to predict events reducing the uncertainty in their environment, therefore increasing welfare (Spruijt et al., 2001). Predictable negative events tend to be less stressful (Weiss, 1970; Galhardo et al., 2011; Piato et al., 2011), since there is a signaling event that enables the organism to anticipate the aversive stimulus. For example, studies in sheep show that by predicting an event (drop of a white and blue panel behind the food container) in an associative scheme with a light cue, lambs showed less startle responses than their counterparts in an unpredictable treatment (Greiveldinger et al., 2007). Some studies suggest that it is not the anticipation of the negative stimulus, but the absence of the signal that predicts the aversive event that makes predictable aversive events less stressful, since cue absence indicates a safety period (Seligman, 1968; Bassett and Buchanan-Smith, 2007). Fowls trained in classical conditioning (conditioned stimulus—CS: music; unconditioned stimulus—US: food), showed comfort behaviors, such as peening and wing flapping, in anticipation of food (Zimmerman et al., 2011). Moreover, rats trained in a Pavlovian conditioning paradigm, through the association of a tone (CS) with a food reward (US), showed an increase in behavioral transitions (hyperactive) during the 3 min interval between CS and US, as a result of anticipation of food (van den Bos et al., 2003). Furthermore, the same study suggests that the conditioned response in cats is different from the one observed in rats in anticipation to the rewarding stimulus (van den Bos et al., 2003). Similarly, studies in halibut showed that delay and trace conditioning elicited different conditioned responses, depending on the temporal presentation of CS and US (Nilsson et al., 2010). Intrinsic pleasantness component determines the valence (positive or negative) of the event (Ellsworth and Scherer, 2003). Organisms’ approach/withdrawal responses have been used to assess pleasantness of events (Xu et al., 2007; Al-Imari and Gerlai, 2008; Millot et al., 2014), with approach and withdrawal referring, respectively, to positive/pleasant and aversive/unpleasant. In an associative learning study, zebrafish learned to swim from a light compartment (CS) to a darker compartment to avoid an electric shock, a withdrawal response that indicates unpleasantness towards the aversive event (Xu et al., 2007). Furthermore, the same species was also able to associate a visual cue (red card) with a rewarding stimulus (sight of a shoal), so that when tested in the presence of the red card itself, fish spent more time in the proximity of the visual cue than animals in the unpaired group, where no association between the red card and the shoal was established (Al-Imari and Gerlai, 2008). The proximity of zebrafish to the visual cue associated to the shoal demonstrates the pleasantness associated with conspecifics presence and suggests the shoal as a rewarding stimulus.

Other appraisal components determine to what extent an animal can evaluate the implications of a situation and control or adapt to it—coping component (Scherer, 2001; Ellsworth and Scherer, 2003). One can cope with a stimulus by controlling it (controllability) or, in cases when control is not possible, by adjusting to it (adjustment) (Scherer, 2001). A pioneer study conducted in rats showed that animals choose preferentially an operant-obtained food reward instead of a free reward, indicating that control itself can be rewarding (Overmier et al., 1980). This phenomenon is also described in the literature as contrafreeloading (reviewed in Inglis et al., 1997). Additionally, controlling an event can be perceived as less stressful, as demonstrated by Greiveldinger et al. (2009) where lambs that controlled (learning an operant task) an aversive event that would prevent access to food, were more willing to enter the test arena and eat there. Conversely, when exerting control is not possible, animals can cope by adapting to the situation (Désiré et al., 2002). For instance, a study showed that rats which performed a coping response to escape or avoid an electrical shock, by jumping up onto a platform when the aversive stimulus was delivered, developed less physiological symptoms (weight loss and gastric lesions) of stress than the group where coping was not possible (Weiss, 1968).

Appraisal components mentioned above operate at different levels of processing (Leventhal and Scherer, 1987), namely: (1) sensorimotor—that includes automatic processes and implies innate sensory-motor programs; (2) schematic—an automatic mechanism that involves schemata matching processes, which integrates sensory-motor and cues (e.g., visual) signaling specific affective situations; and (3) conceptual—complex processing that requires the evaluation of stimulus implications and translates into a problem solving ability that promotes the adjustment to the new situation. If the stimulus is novel (novelty), its processing requires innate sensorimotor mechanisms, since there is no information previously stored that needs to be integrated in the response (Désiré et al., 2002). On the contrary, if the event is familiar, predictable or pleasant, the animal has to combine past knowledge of the situation with current information, in a schematic level of processing (Désiré et al., 2002). Finally, the coping component, which is based on the assessment of stimulus implications, allows the animal to control or adapt to the event, involving a problem-solving ability, characteristic of the conceptual level of processing (Désiré et al., 2002).

Although animal research suggests the existence of appraisal components in animals (Wong et al., 2010; Galhardo et al., 2011; Piato et al., 2011), studies verifying the contribution of several components to the evaluation of the same stimulus are still missing in non-human animals. Several experiments by Greiveldinger and colleagues (Greiveldinger et al., 2007, 2009, 2011) suggest the presence of multicomponent appraisal in lambs by validating several appraisal components: suddenness, predictability, controllability and discrepancy from expectations. By using the same stimulus (feeding event), Greiveldinger and co-workers saw that lambs were able to predict food delivery (Greiveldinger et al., 2007), to control an aversive event in order to have access to food (Greiveldinger et al., 2009), to memorize the amount of food given and form expectations about the load of food that was going to be supplied (Greiveldinger et al., 2011). Taken together, these studies seem to indicate a distinct contribution of different appraisal components, operating at different levels of processing (Scherer, 2001), to the evaluation of the same event. While predictability (Greiveldinger et al., 2007) is an appraisal component operating on the schematic level of processing, controllability (Greiveldinger et al., 2009) and discrepancy from expectations (Greiveldinger et al., 2011) may imply problem solving abilities, hence providing an example of a more elaborate processing of environmental information in non-human animals.

It seems reasonable to state that approaches like the one Greiveldinger and colleagues followed, should be used when it comes to attest the presence of appraisal in animals. Moreover, appraisal research in non-human animals should pursue approaches that evaluate the presence of different appraisal components to the same event, acting at the sensorimotor, schematic and conceptual levels, leaving behind the tendency to concentrate on simpler components to postulate appraisal in animals.

## Cognitive Bias and Behavioral Flexibility: Implications for Stress Research

Since appraisal allows animals to evaluate stimuli, not only by relying on their intrinsic characteristics but also considering the subject’s internal state (Scherer, 2009), affect-induced alterations of inner state, brought by changes in the environment, can lead to a variety of internal states accountable for biased evaluations of ambiguous events—also known as cognitive biases (Paul et al., 2005; Mendl et al., 2009). Moreover, if appraisal depends on the interaction between stimulus characteristics and internal state, an animal in a negative or positive affective state will tend to evaluate an ambiguous stimulus as more negative or positive, respectively (Mendl et al., 2009).

Several studies have been addressing cognitive biases in animals and how specific affective manipulations influence these processes (Harding et al., 2004; Mendl et al., 2010; Bateson et al., 2011; Rygula et al., 2013). A great part of the literature so far, focused on inducing a negative state by using stressful manipulations (e.g., unpredictable housing, Harding et al., 2004; shaking, Bateson et al., 2011; chronic stress, Rygula et al., 2013). In a seminal paper, Harding and co-workers (Harding et al., 2004) have shown that rats exposed to unpredictable housing conditions exhibited pessimistic evaluations of ambiguous stimuli. In that study, rats were trained to associate a positive tone with a food reward and a negative tone with an aversive white noise, after which they were exposed to an unpredictable housing period. Afterwards, when confronted with non-reinforced tones (ambiguous stimuli), individuals were slower to respond and tended to show fewer responses to ambiguous tones close to the positive tone and to this tone itself, an outcome consistent with a negative state (Harding et al., 2004). More recently, Mendl et al. (2010) showed that dogs that presented more vocalizations and destructive behaviors when left home alone (separation-related behavior) also exhibited a pessimistic bias. These dogs were trained to associate a positive location with food and a negative location with the absence of food (Mendl et al., 2010). When tested for locations in between positive and negative ones (ambiguous locations), dogs with a higher score of separation-related behavior ran slower than the ones with lower scores, confirming that dogs in a more negative state make more negative judgments about ambiguous stimuli (Mendl et al., 2010). Cognitive bias has also been documented in bees (Bateson et al., 2011). Bees were trained to extend their proboscis in response to an odor mixture predictive of a reward (sucrose) (CS+) and to withhold their mouthparts in response to an odor mixture predicting a punishment or a less valuable reward (CS−; Bateson et al., 2011). When tested for novel (ambiguous) odor combinations, shaken bees were more likely to withhold their proboscis to the most similar odor to CS−, conveying evidence for pessimistic bias (Bateson et al., 2011). Finally, a recent study showed that rats displaying the pessimistic trait were more willing to develop stress-induced anhedonia compared to their optimistic counterparts (Rygula et al., 2013). Furthermore, both optimistic and pessimistic animals after being exposed to chronic stress, were more biased to make pessimistic judgments about ambiguous stimuli (Rygula et al., 2013). Rats learned to press a lever to get access to a reward (sucrose) after a positive tone was displayed and learned to press a lever to avoid an electric shock when a negative tone was presented (Rygula et al., 2013). After training, rats were tested with an intermediate tone to screen them for cognitive bias, and pessimistic animals were more prone to press the negative cued lever, whereas the optimistic individuals were more willing to press the positive cued lever (Rygula et al., 2013). Interestingly, rats belonging to the control group showed pessimistic and optimist judgments over time, which shows that cognitive bias, as in humans, reunites characteristics both of a trait and of a transient state (Kluemper et al., 2009; Rygula et al., 2013).

**Figure 1:**
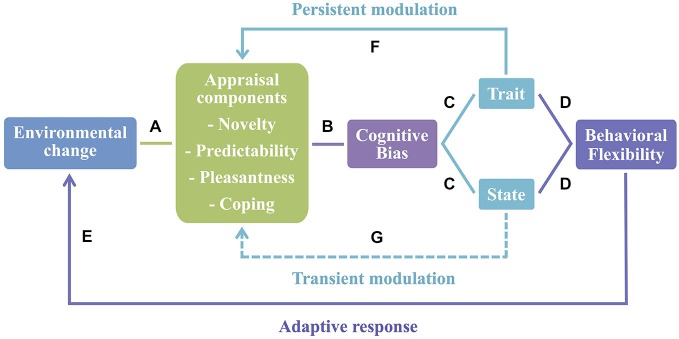
**Appraisal as the basis of behavioral flexibility. (A)** Animals evaluate environmental changes using an appraisal process that comprises a set of stimulus evaluation checks (here represented by appraisal components). **(B)** Once changes in the environment induce alterations in affective states of animals, cognitive bias arises in contexts of stimulus ambiguity. **(C)** Cognitive bias may be seen either as a state or a trait. As a state it may change depending on the situation or contextual factors—within-individual variability; and as a trait it is consistent over time with different individuals exhibiting a specific phenotype—between-individual variability (e.g., optimistic/pessimistic). **(D)** Both mechanisms are characteristic of behavioral flexibility. **(E)** These behavioral flexibility processes promote an adaptive response to new environmental changes that may occur. **(F)** Conversely, the appraisal process may be subject to a long-lasting modulation by cognitive bias as a trait, and short-term effects **(G)** are expected when cognitive bias occurs as a state.

Based on these findings, we suggest that cognitive bias can be conceptualized as a plastic trait (i.e., a behavioral trait that is susceptible to environmental variation, Dingemanse et al., 2010). Thus, cognitive bias in animals can be interpreted as an open program, since it enables the integration of additional inputs and is subject to environmental modulation over time (Mayr, 1974). Moreover, since animals are exposed to rapid and constant changes in the environmental conditions, cognitive bias can be used as an adjustment to a new context and considered a case of behavioral flexibility. It has been suggested that behavioral flexibility is characterized by within and between-subjects variability mechanisms (Piersma and Drent, 2003; Taborsky and Oliveira, 2012) and both of these mechanisms seem to be present in the case of cognitive bias. As a state, it may change depending on the situation or contextual factors (within-subjects variability). Once individuals exhibit a phenotype (e.g., optimistic/pessimistic) consistent over time (trait), it may act as a mechanism of between-subjects variability (see Figure 1, for a model linking appraisal and behavioral flexibility in animals).

By being open to environmental modulation, optimistic and pessimistic phenotypes may differ in the resilience they confer to the individual in stressful situations. Assessing the stress resilience of optimistic and pessimistic phenotypes seems especially relevant for the study of stress related disorders (SRD; Kalisch et al., 2014). In fact, patients suffering from SRD often evaluate ambiguous cues as negative, hence frequently leading to negative states (Kalisch et al., 2014). Data in human studies show that individuals with severe depression, when making predictions about their future, tend to expect things to be worse than they turn out to be—pessimistic bias (Strunk et al., 2006). In rodents, studies have emphasized the influence of depressive-like states (Enkel et al., 2010) in cognitive biases by showing that both congeniality helpless rats (a genetic model of depression) and rats in a noradrenergic-glucocorticoid challenge (stress-related changes in endogenous neuromodulation) showed pessimistic bias towards ambiguous stimuli. Moreover, Rygula et al. (2013) demonstrated that anhedonia occurs faster and lasts longer in pessimistic rats, confirming that both traits are different in their vulnerability to stress-induced depressive symptoms. We argue that some biases (optimistic) may be more stress resilient and that cognitive bias may be an effective way of assessing resilience and its neural mechanisms in animals. By focusing on cognitive bias paradigms it would be possible to assess how negative or positive states/traits influence one’s evaluation of ambiguous stimuli and which individuals are more stress resilient (i.e., individuals in negative states are less resilient to aversive events) (Rygula et al., 2013). Furthermore, screening animals for pessimistic and optimistic phenotypes may be helpful in the field of antidepressant drugs research, since pessimistic and optimistic animals may diverge in their response to these antidepressants (Rygula et al., 2013).

## Concluding Remarks

The evidence reviewed in this article supports the hypothesis that appraisal is a key mechanism for behavioral flexibility, allowing the adjustment of the organism to complex and changing environments. In this regard, the study of cognitive bias has shown how affect manipulations induce alterations in judgment biases, with a large body of work suggesting that negative affect manipulations lead to negative states (pessimistic). Based on the research reviewed, we also suggest that cognitive bias could be interpreted as a trait open to environmental modulation (Dingemanse et al., 2010), that confers alternative strategies for dealing with stressful situations (Rygula et al., 2013).

Although we can identify an emerging literature in appraisal processes in animals, most research has focused on stimulus relevance (novelty, predictability, pleasantness). Studies focusing on other components are required for a better understanding of the mechanisms involved in the appraisal process and the proximate causes of behavior. Furthermore, research approaches that evaluate the presence of different appraisal components (acting at the different levels of processing) should be considered, since one can only demonstrate appraisal by proving the existence of components that use simple and complex levels of processing.

## Conflict of Interest Statement

The authors declare that the research was conducted in the absence of any commercial or financial relationships that could be construed as a potential conflict of interest.
